# Supplementary material for: Multiple C2 domains and transmembrane region proteins (MCTPs) tether membranes at plasmodesmata
Source: EMBO Rep. 2019 Jul 9;20(8):e47182. doi: 10.15252/embr.201847182 (PMC6680132; doi:10.15252/embr.201847182)
Supplement: Supplementary file 1 — Appendix [file EMBR-20-e47182-s001.pdf]

Table of content

**Appendix Figure S1**	**2-3**
**Appendix Figure S2**	**4-5**
**Appendix Figure S3**	**6**
**Appendix Figure S4**	**7-8**
**Appendix Figure S5**	**9**
**Appendix Figure S6**	**10-11**
**Appendix Figure S7**	**12-13**
**Appendix Figure S8**	**14**
**Appendix Figure S9**	**15**
**Appendix Figure S10**	**16**
**Appendix Figure S11**	**17-18**
**Appendix Figure S12**	**19-20**
**Appendix Figure S13**	**21-22**
**Appendix Figure S14**	**23**
**Appendix Table S1**	**24-25**
**Appendix Table S2**	**26**

Appendix Figure S1

a

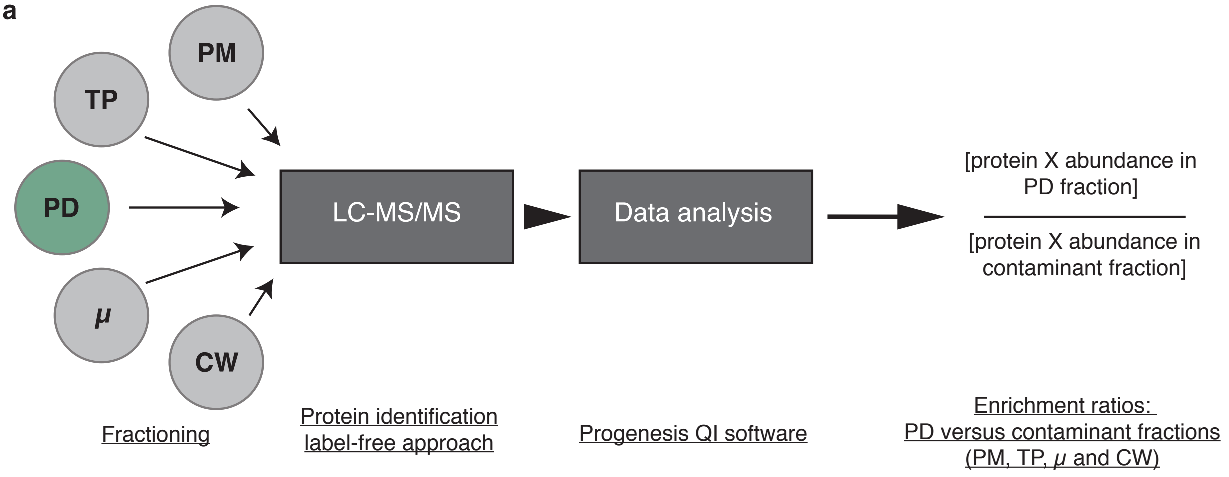

b

Description	Abundance	Enrichment ratios				ER proteomes		PD association references
		PD/PM	PD/TP	PD/μ	PD/CW	1.	2.	
**Multiple C2 domains and Transmembrane region Protein 4,10,14 (MCTP4,10,14)**	**2093561645**	**351,0**	**223,6**	**360,1**	**70,2**	×	×	—
Beta-1-3-glucanase (AtBG_PAPP)	1638015771	164,0	247,2	580,8	45,0			3.
**Multiple C2 domains and Transmembrane region Protein 6,9 (MCTP6,9)**	**776007012**	**315,5**	**115,1**	**285,3**	**61,7**			—
Plasmodesmata callose-binding protein 1 (PDCB1)	328259264	219,2	1052,3	623,0	48,0			4.
Plasmodesmata-located protein 1 (PDLP1)	311480268	309,0	119,0	307,6	46,4			5.
Glucan synthase-like 12 (CALS3)	257637656	14,5	56,4	67,3	65,2			6.
O-Glycosyl hydrolases family 17 protein (beta1-3 glucanase, PdBG2)	232481254	26,9	73,3	89,6	48,4			7.
Plasmodesmata-located protein 6 (PDLP6)	159384568	193,7	126,1	637,9	52,3			5.
Plasmodesmata callose-binding protein 3 (PDCB3)	100145419	101,4	63,2	76,5	46,8			4.
Plasmodesmata callose-binding protein 4 (PDCB4)	79562157	107,9	133,1	129,2	47,5			4.
O-Glycosyl hydrolases family 17 protein (beta1-3 glucanase, PdBG3)	71917917	32,9	204,2	237,4	59,5			7.
Plasmodesmata-located protein 3 (PDLP3)	71730983	251,4	90,8	325,4	60,7			5.
O-Glycosyl hydrolases family 17 protein (beta1-3 glucanase, PdBG1)	65897722	42,7	148,4	287,3	52,3			7.
Tetraspanin 3 (TET3)	47760446	65,3	102,4	242,7	51,0			10.
LysM domain-containing GPI-anchored protein 2 (LYM2)	40630549	2,7	18,3	10,3	35,9			8.
Plasmodesmata-located protein 2 (PDLP2)	38475248	172,0	78,7	74,5	44,9			5.
Callose synthase 1 (CALS1, GSL6)	29840182	14,0	39,5	40,0	69,2			9.
Probable receptor-like protein kinase	25515183	4,9	29,4	23,8	58,0			10.
**Multiple C2 domains and Transmembrane region Protein 16 (MCTP16)**	**23482273**	**59,7**	**33,5**	**126,7**	**34,9**	×	×	—
**Multiple C2 domains and Transmembrane region Protein 3,7 (MCTP3,7)**	**20441820**	**47,5**	**44,3**	**96,9**	**81,7**	×	×	—
**Multiple C2 domains and Transmembrane region Protein 15 (MCTP15, QUIRKY, QKY)**	**15148937**	**79,0**	**47,9**	**82,9**	**73,1**			11.
**Multiple C2 domains and Transmembrane region Protein 5 (MCTP5)**	**9974540**	**102,5**	**516,4**	**171,4**	**152,6**			—
Receptor kinase 3 (SD18)	8493304	14,4	6,1	7,2	34,6			10.
Leucine-rich repeat protein kinase family protein (SUB)	6660962	31,8	40,7	68,3	55,6			11.
Plasmodesmata-located protein 8 (PDLP8)	2101866	365,8	32,1	214,6	48,5			5.

1. Nikolovski, N. et al. Putative glycosyltransferases and other plant golgi apparatus proteins are revealed. Plant Physiol. 160, 1037–1051 (2012).
2. Dunkley, T. P. J. et al. Mapping the Arabidopsis organelle proteome. PNAS 103, 6518–6523 (2006).
3. Levy, A., Erlanger, M., Rosenthal, M. & Epel, B. L. A plasmodesmata-associated beta-1,3-glucanase in Arabidopsis. Plant J. 49, 669–682 (2007).
4. Simpson, C., Thomas, C., Findlay, K., Bayer, E. & Maule, A. J. An Arabidopsis GPI-anchor plasmodesmal neck protein with callose binding activity and potential to regulate cell-to-cell trafficking. Plant Cell 21, 581–594 (2009).
5. Thomas, C. L., Bayer, E. M., Ritzenthaler, C., Fernandez-Calvino, L. & Maule, A. J. Specific targeting of a plasmodesmal protein affecting cell-to-cell communication. PLoS Biol. 6, 0180–0190 (2008).
6. Vatén, A. et al. Callose biosynthesis regulates symplastic trafficking during root development. Dev. Cell 21, 1144–1155 (2011).
7. Benitez-Alfonso, Y. et al. Symplastic intercellular connectivity regulates lateral root patterning. Dev. Cell 26, 136–147 (2013).
8. Faulkner, C. et al. LYM2-dependent chitin perception limits molecular flux via plasmodesmata. Proc. Natl. Acad. Sci. U. S. A. 110, 9166–70 (2013).
9. Cui, W. & Lee, J.-Y. Arabidopsis callose synthases CalS1/8 regulate plasmodesmal permeability during stress. Nat. Plants 2, 16034 (2016).
10. Fernandez-Calvino, L. et al. Arabidopsis plasmodesmal proteome. PLoS One 6, e18880 (2011).
11. Vaddepalli, P. et al. The C2-domain protein QUIRKY and the receptor-like kinase STRUBBELIG localize to plasmodesmata and mediate tissue morphogenesis in Arabidopsis thaliana. Development 141, 4139–4148 (2014).

Appendix Figure S1.

MCTP members are highly enriched in the *Arabidopsis* plasmodesmata core proteome.

(a) Label-free quantitation strategy was used to determine the relative abundance of proteins in the plasmodesmata (PD) fraction *versus* contaminant subcellular fractions namely, the PM, total extract (TP), microsomes (μ) and cell wall (CW).

(b) Selected set of proteins from the plasmodesmata core proteome (see Supplementary Table1 for the complete list) showing the abundance and enrichment ratios of known plasmodesmal proteins (reference to published papers is indicated below the table) and MCTP members (in bold). MCTP members are present in the plasmodesmal core proteome being both abundant and highly enriched (from 47.5- to 351-folds compared to the PM) similar to known plasmodesmata proteins. Please note that in some cases, the identified peptides did not permit unambiguous identification of MCTP isoforms due to high sequence homology between several members. The different shades (light to dark) of brown represent different enrichment levels (0-10; 10-20; 20-100 and above 100).

Appendix Figure S2

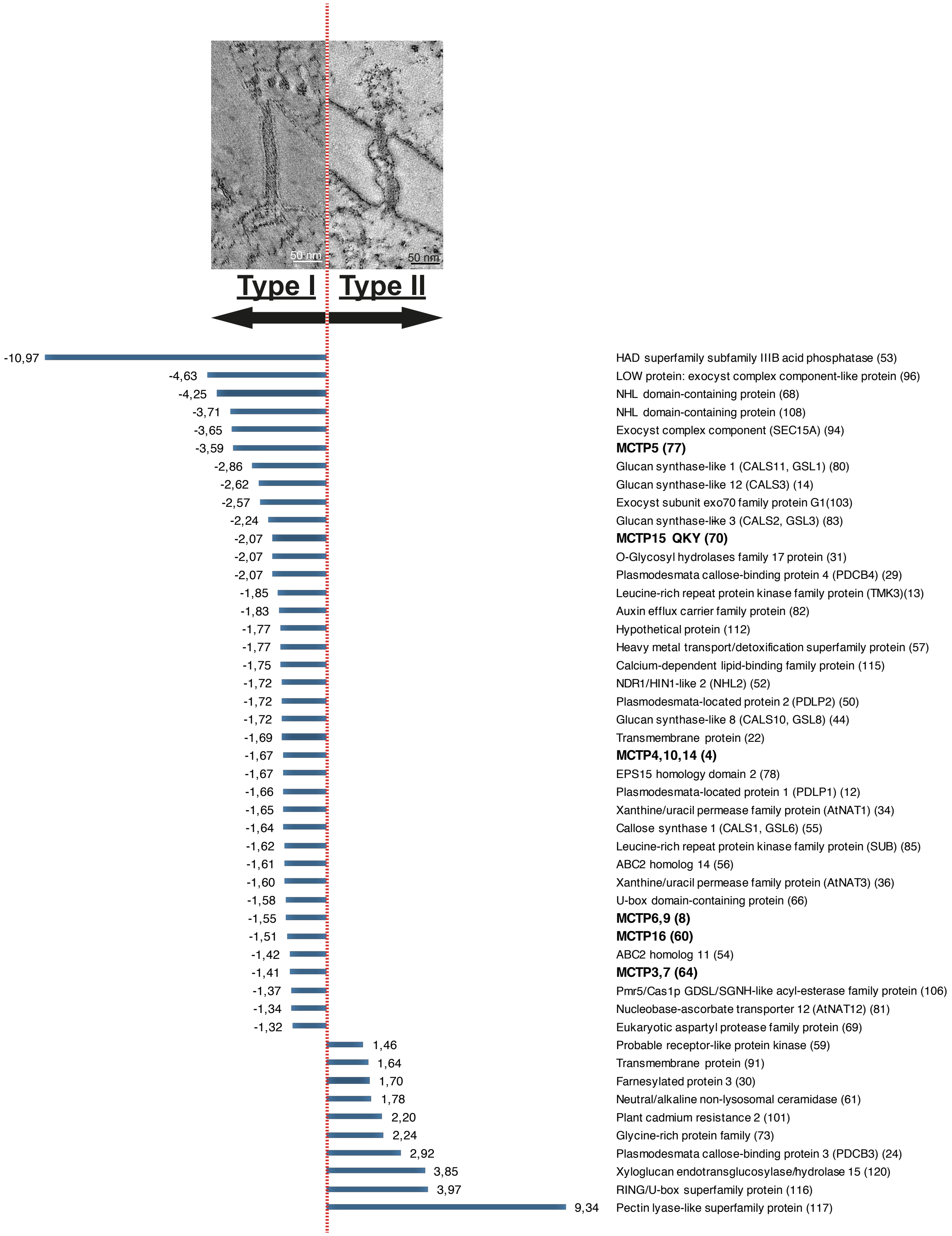

Appendix Figure S2.

Differential abundance of core *Arabidopsis* plasmodesmal proteins in type I (four day old cultured cells) versus type II (seven day old cells) plasmodesmata.

In *Arabidopsis* cultured cells, transition from type I to type II plasmodesmata is associated with a change in ER-PM contact site architecture, from very tight contact (~3 nm) with no visible cytoplasmic sleeve (type I) to larger ER-PM distance (10 nm to more) with an electron lucent cytosolic sleeve and sparse spoke-like elements (type II) [1]. We analysed the plasmodesmata proteome from four days old cultured cells where type I plasmodesmata represent 70% of the total plasmodesmata population and at seven days where this proportion is reversed and type II become predominant [1]. Results show that 47 proteins from the plasmodesmata core proteome are differentially enriched at either type I or type II plasmodesmata, including all members of MCTPs (in bold), which are more abundant (1.4 to 3.6 folds) in type I plasmodesmata. Numbers in brackets correspondent to the protein numbering in Suppl. Table 1.

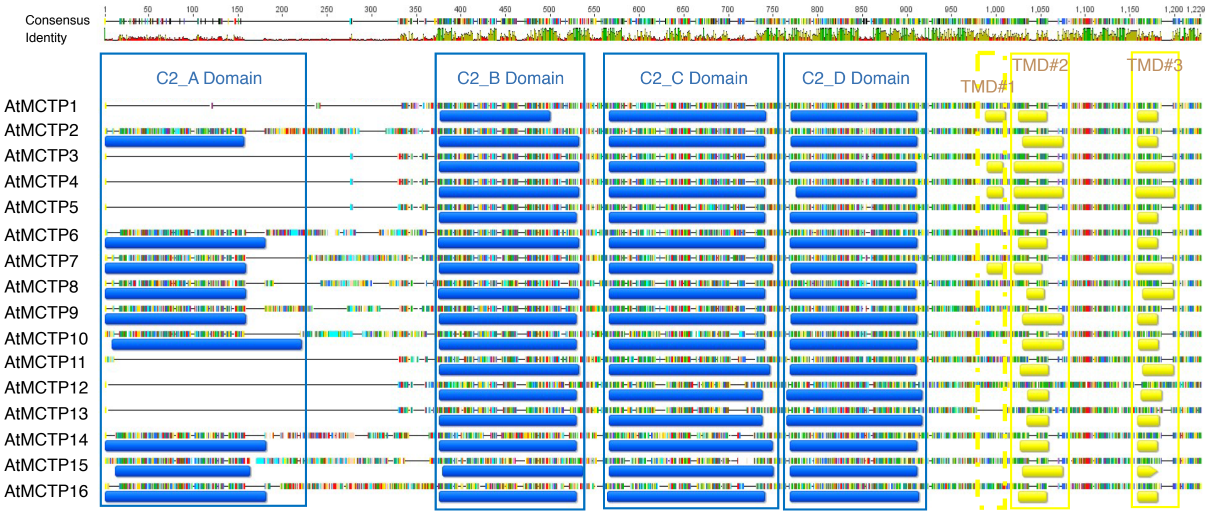

Appendix Figure S3.

Domain organisation of the *Arabidopsis* MCTP protein family.

Alignment of the 16 MCTP proteins of *A. thaliana.* C2 domains are represented in blue and transmembrane domains (TMD) in yellow. Each coloured vertical bars represents specific amino acid. The consensus sequence and the percentage of identity are represented on the top of the alignment. Note that for every MCTP member the C2 domains were individually delimitated using a combination of prediction methods (see M&M for details).

Appendix Figure S4

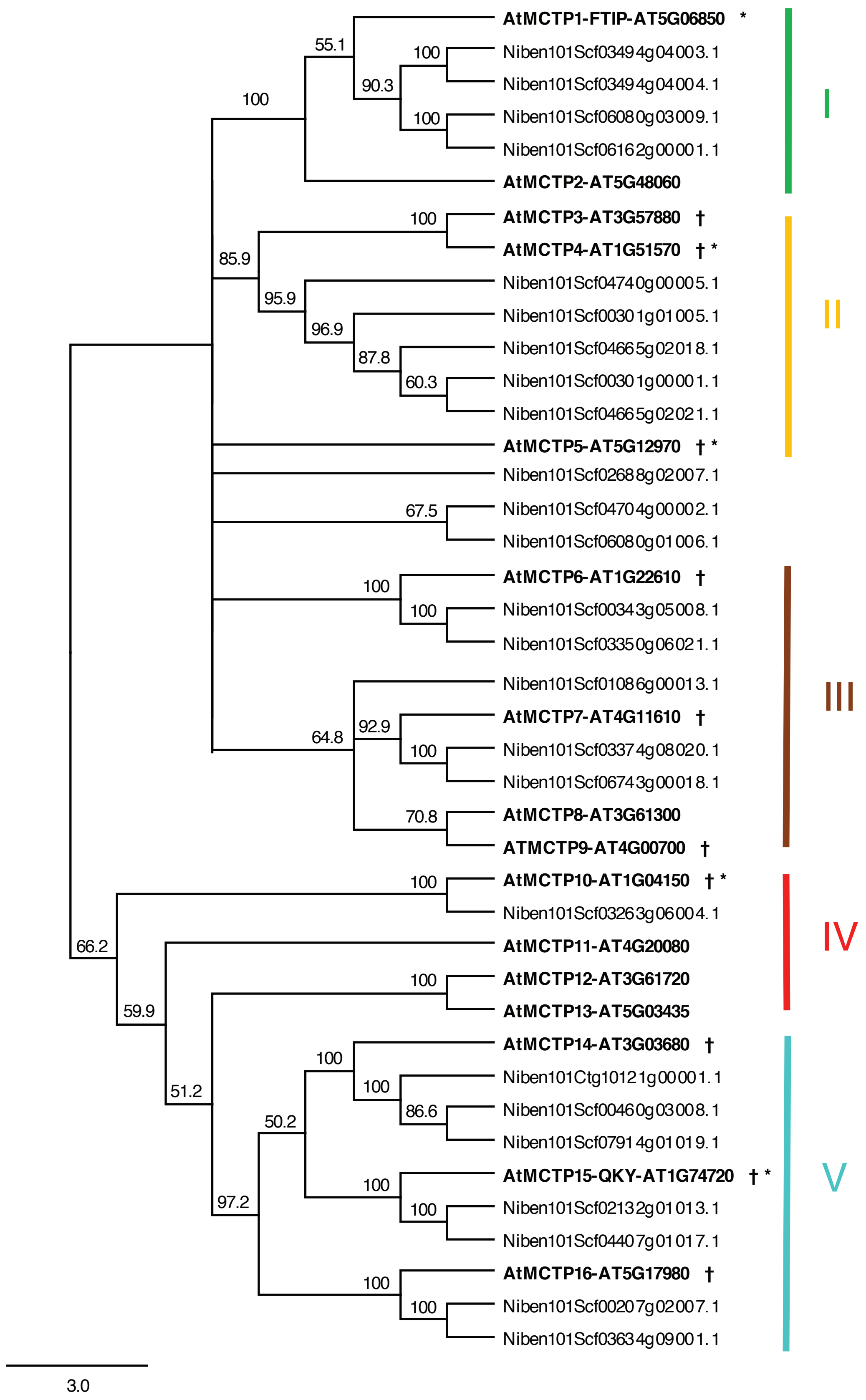

Appendix Figure S4.

Phylogenetic tree of *A. thaliana* and *N. benthamiana* MCTP proteins. Amino acid sequences of MCTP family from *A. thaliana* and *N. benthamiana* were aligned with CLUSTALW [2]. The resulting alignment was adjusted manually and used to construct an unrooted phylogenetic tree using the neighbour-joining algorithm with Geneious 8.0.5 (https://www.geneious.com). Bootstrap values for 1000 re-samplings are shown on each branch. † indicates the MCTP members enriched in the plasmodesmata proteome and * indicates the MCTP members enriched in type I plasmodesmata. The five clades defined in Liu *et al.* 2017 [3] are indicated from I to V.

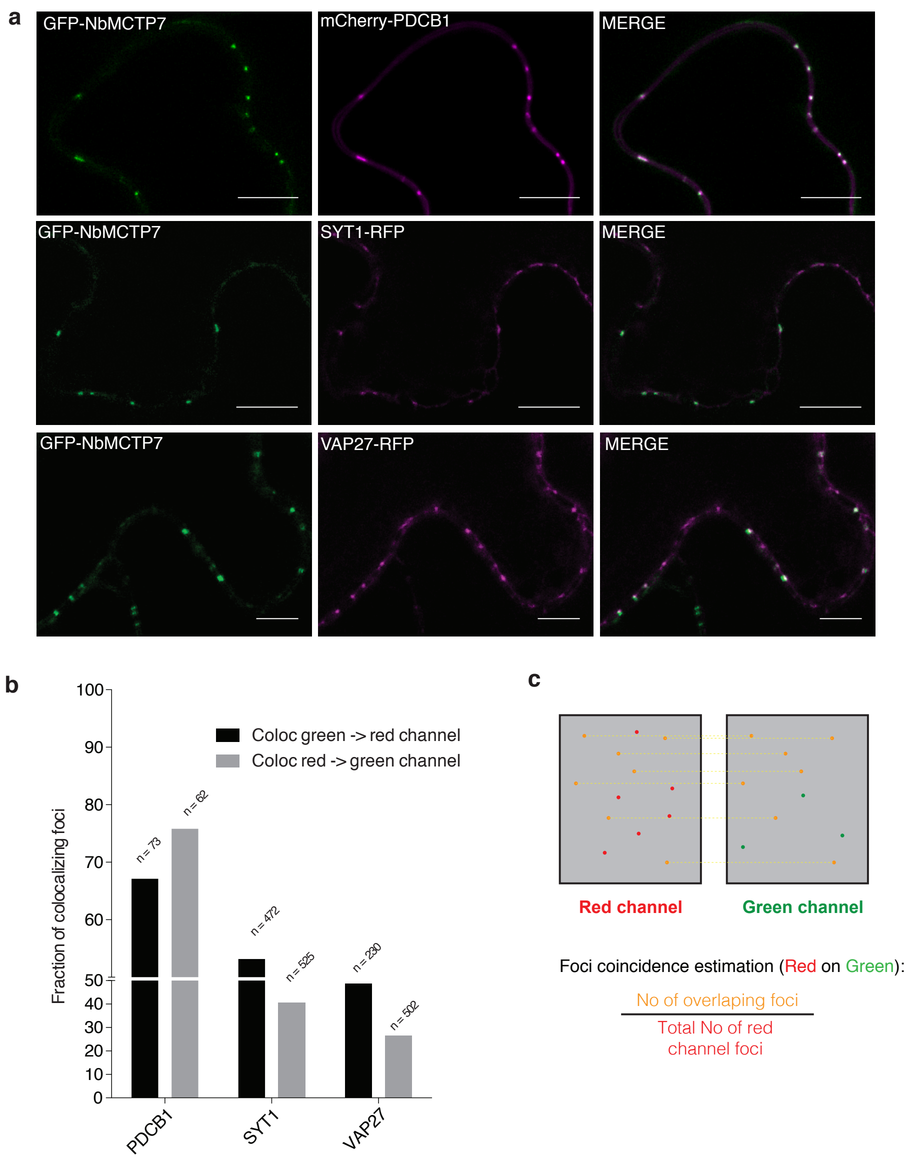

Appendix Figure S5.

NbMCTP7 only partially co-localise with peripheral ER-PM contact sites.

(a) Co-localisation between GFP-NbMCTP7 with mCherry-PDCB1 and two well-established markers of peripheral ER-PM contact sites, VAP27.1 [4] and SYT1 [5,6], in *N. benthamiana* epidermal cells visualised by confocal microscopy. Scale bars, 10 μm.

(b) Plot of the coincidence ratios. “Coloc green -> red channel” depicts the proportion of foci in the green channel overlapping with foci of the red channel over the total number of foci in the green channel. “Coloc red -> green channel” depicts this same proportion but of the red foci over the green foci. Coefficients range from 0 (complete exclusion) to 100% (complete colocalization of all foci). N indicated is the number of foci counted over 10 images of a given condition acquired over multiple co-expression/imaging sessions.

(c) Cartoon schematic on how the Coincidence ratio is calculated.

Appendix Figure S6

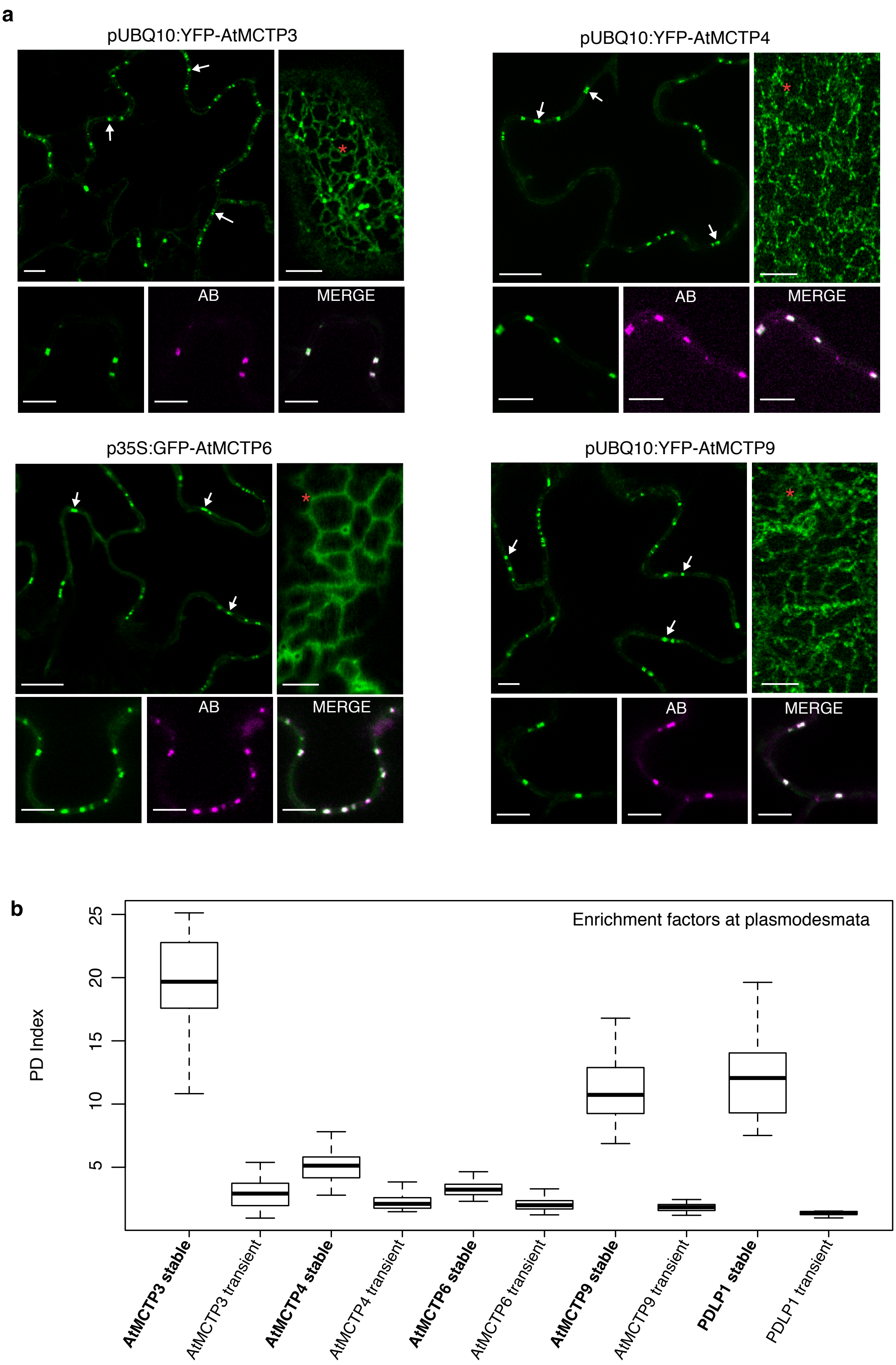

Appendix Figure S6.

Subcellular localisation pattern of AtMCTP3, AtMCTP4, AtMCTP6 and AtMCTP9 when stably expressed in *Arabidopsis*.

(a) Subcellular localisation of pUBQ10:YFP-AtMCTP3, pUB10:YFP-AtMCTP4, 35S:GFP-MCTP6 and pUB10:YFP-AtMCTP9 in transgenic *Arabidopsis* epidermal cells showing typical plasmodesmata punctate pattern at the cell periphery (white arrows) and reticulated ER pattern at the cell surface (red stars). Plasmodesmal localisation was confirmed by aniline blue (AB) co-staining. Scale bars, 5 µm.

(b) Plasmodesmata (PD) index of *Arabidopsis* MCTPs and 35S:PDLP1-RFP when either stably expressed transgenic Arabidopsis, or transiently expressed in *N. benthamiana*, showing consistently increased plasmodesmata association in transgenic lines. Three biological replicates were analysed. In the box plot, median value is represented by horizontal line, values between quartil 1 to 3 are represented by box ranges, minimum and maximum values are represented by error bars.

Appendix Figure S7

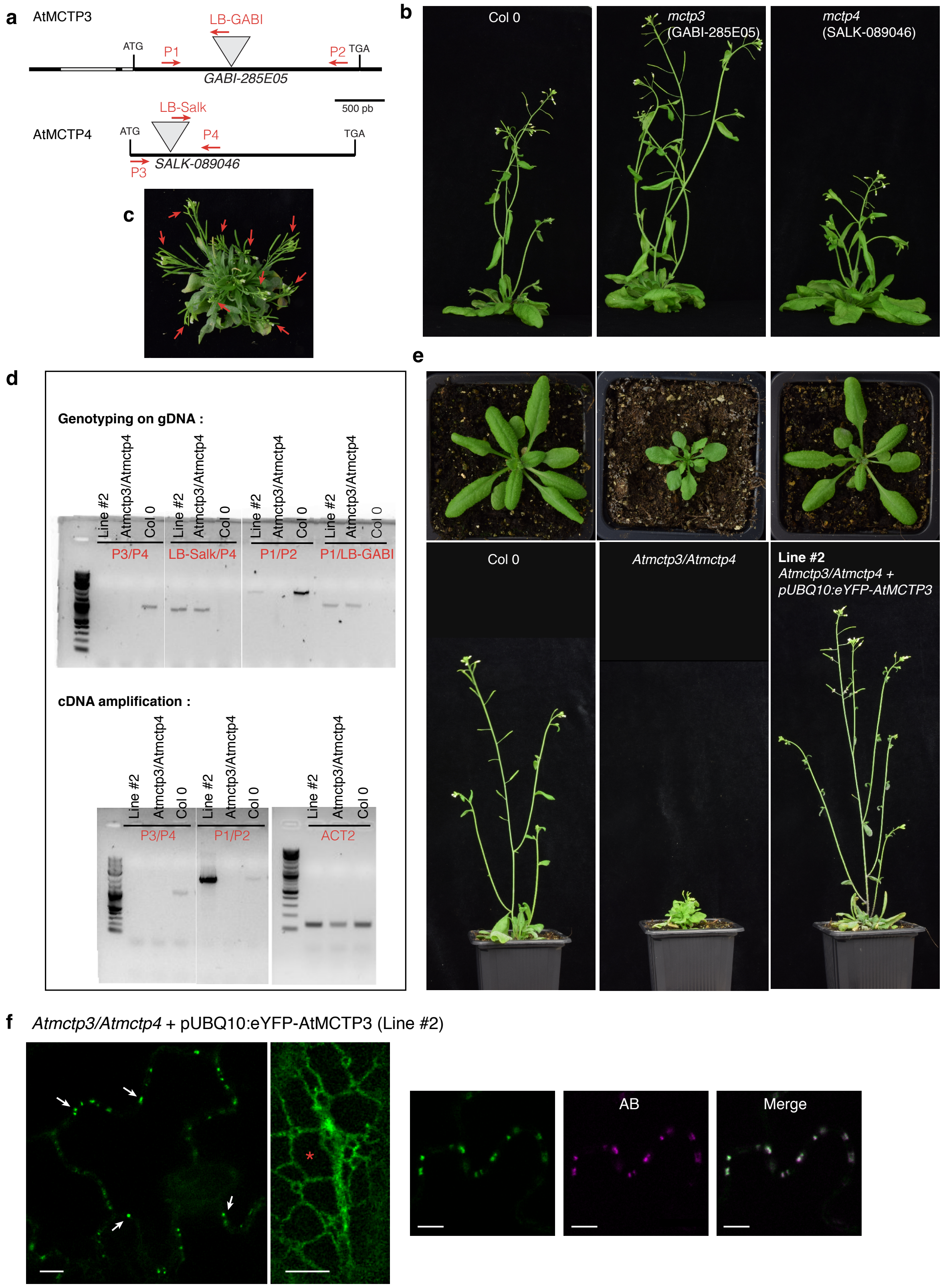

Appendix Figure S7

YFP-AtMCTP3 expression complements *Atmctp3/Atmctp4* loss-of-function double mutant**.**

(a) Schematic representation of T-DNA insertions in AtMCTP3 and AtMCTP4. LB, left border. In red, primers used for genotyping and RT-PCR. (b) Inflorescence stage of Col 0 *Atmctp3* (GABI-285E05) and *Atmctp4* (SALK-089046). (c) *Atmcpt3/Atmctp4* double mutant shows multiple inflorescences (red arrows). (d) Top, genotyping of *Atmctp3/Atmctp4* complemented with pUBQ10:YFP-AtMCTP3 (Line #2), *Atmctp3/Atmcpt4* double mutant and Col-0, showing the presence of AtMCTP3 and AtMCTP4 T-DNA inserts. Bottom, RT-PCR analysis of AtMCTP3, AtMCTP4 and Actin2 (ACT2) on cDNA extracted from complemented *Atmctp3/Atmctp4* line #2, *Atmctp3/Atmctp4* double mutant and Col-0 showing the absence of full-length AtMCTP4 transcripts and the over expression of AtMCTP3. (e) Rosette and inflorescence stage of Col 0, *Atmctp3/Atmctp4* double mutant and the complemented line #2. (f) Subcellular localization of YFP-AtMCTP3 in the complemented *Atmctp3/Atmctp4* line visualised by confocal microscopy. Arrows indicate plasmodesmata pietfields. * indicates ER strand. Scale bar, 5 µm.

AtMCTP3 protein sequence

MQRPPPEDFS LKETRPHLGG GKLSGDKLTS TYDLVEQMQY LYVRVVKAKE LPGKDMTGSC DPYVEVKLGN YKGTTRHFEK
KSNPEWNQVF AFSKDRIQAS FLEATVKDKD FVKDDLIGR**V VFDLNEVPK**R VPPDSPLAPQ WYRLEDRKGD KVKGELMLAV
WFGTQADEAF PEAWHSDAAT VSGTDALANI RSKVYLSPKL WYLRVNVIEA QDLIPTDK**QR YPEVYVK**AIV GNQALRTRVS
QSRTINPMWN EDLMFVAAEP FEEPLILSVE DRVAPNKDEV LGRCAIPLQY LDRRFDHKPV NSRWYNLEKH IMVDGEKKET
KFASRIHMRI CLEGGYHVLD ESTHYSSDLR PTAKQLWKPN IGVLELGILN ATGLMPMKTK DGRGTTDAYC VAKYGQKWIR

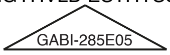

TRTIIDSFTP RWNEQYTWEV FDPCTVVTVG VFDNCHLHGG EKIGGAKDSR IGKVRIRLST LETDRVYTHS YPLLVLHPNG
VKKMGEIHLA VRFTCSSLLN MMYMYSQPLL PKMHYIHPLT VSQLDNLRHQ ATQIVSMRLT RAEPPLRKEV VEYMLDVGSH
MWSMRRSKAN FFRIMGVLSG LIAVGKWFEQ ICNWKNPITT VLIHLLFIIL VLYPELILPT IFLYLFLIGI WYYRWRPRHP
PHMDTRLSHA DSAHPDELDE EFDTFPTSRP SDIVRMRYDR LRSIAGRIQT VVGDLATQGE RLQSLLSWRD PRATALFVLF
CLIAAVILYV TPFQVVALCI GIYALRHPRF RYKLPSVPLN FFRRLPARTD CML

Appendix Figure S8

AtMCTP3 protein sequence with in green the unique peptides identified in proteomics (Fig. 4) and position of the T-DNA.

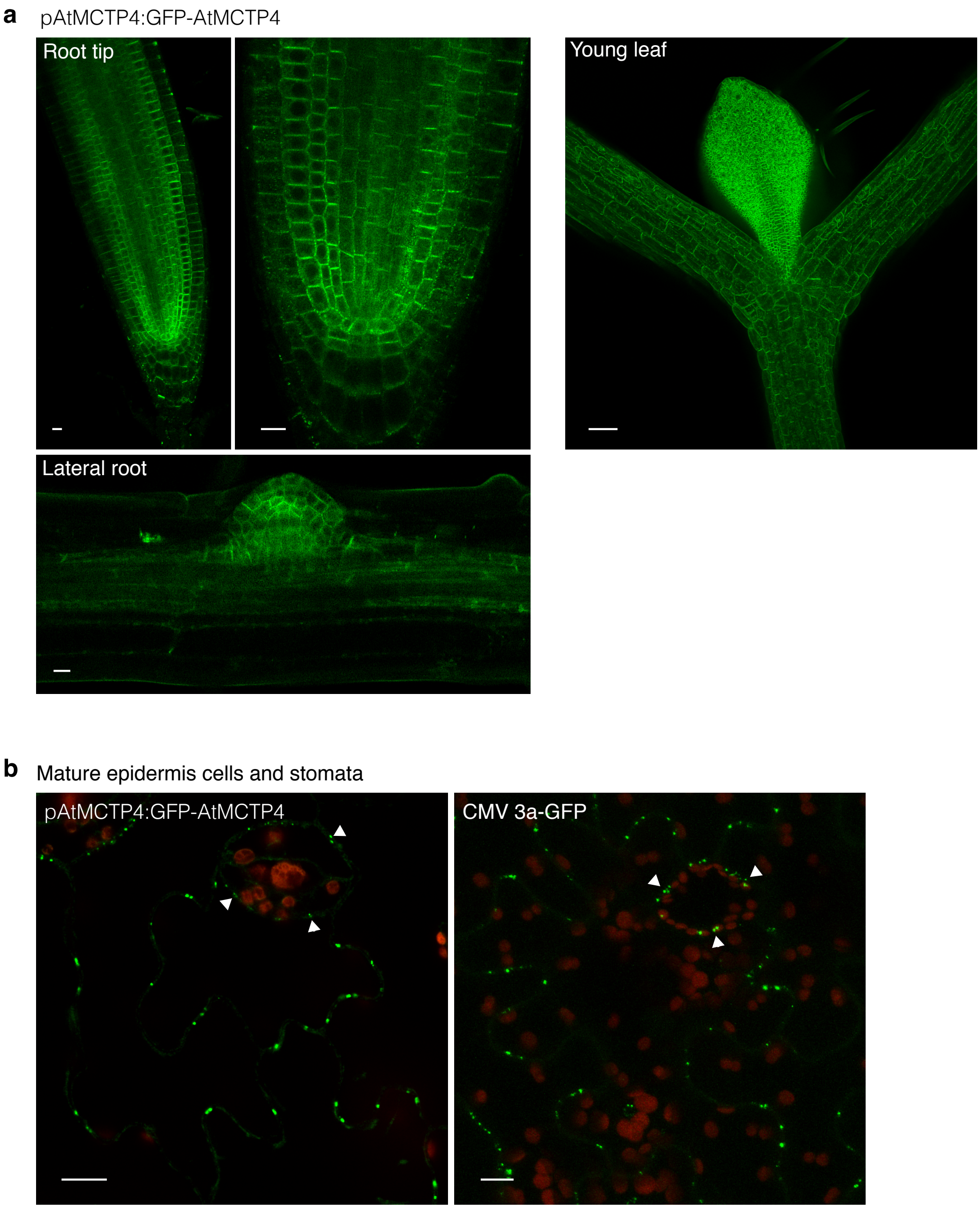

Appendix Figure S9

Overview of *Arabidopsis* seedlings showing the expression pattern of GFP-AtMCTP4 protein under native promoter visualised by confocal microscopy. (a) GFP-AtMCTP4 is strongly expressed in young leaf primordia, in root tip and lateral root of *Arabidopsis*. Scale bars, root tip and lateral root 10 µm; young leaf 100 µm. (b) Localisation pattern of GFP-AtMCTP4 (in *Arabidopsis thaliana*) and Cucumber mosaic virus movement protein CMV 3a-GFP (in Nicotiana *benthamiana*) in leaf epidermal cells is similar. Both proteins display a characteristic plasmodesmata-punctate localisation pattern at the cell periphery and stomata. Arrowhead indicate punctate signal in stomata. Scale bar, 10µm.

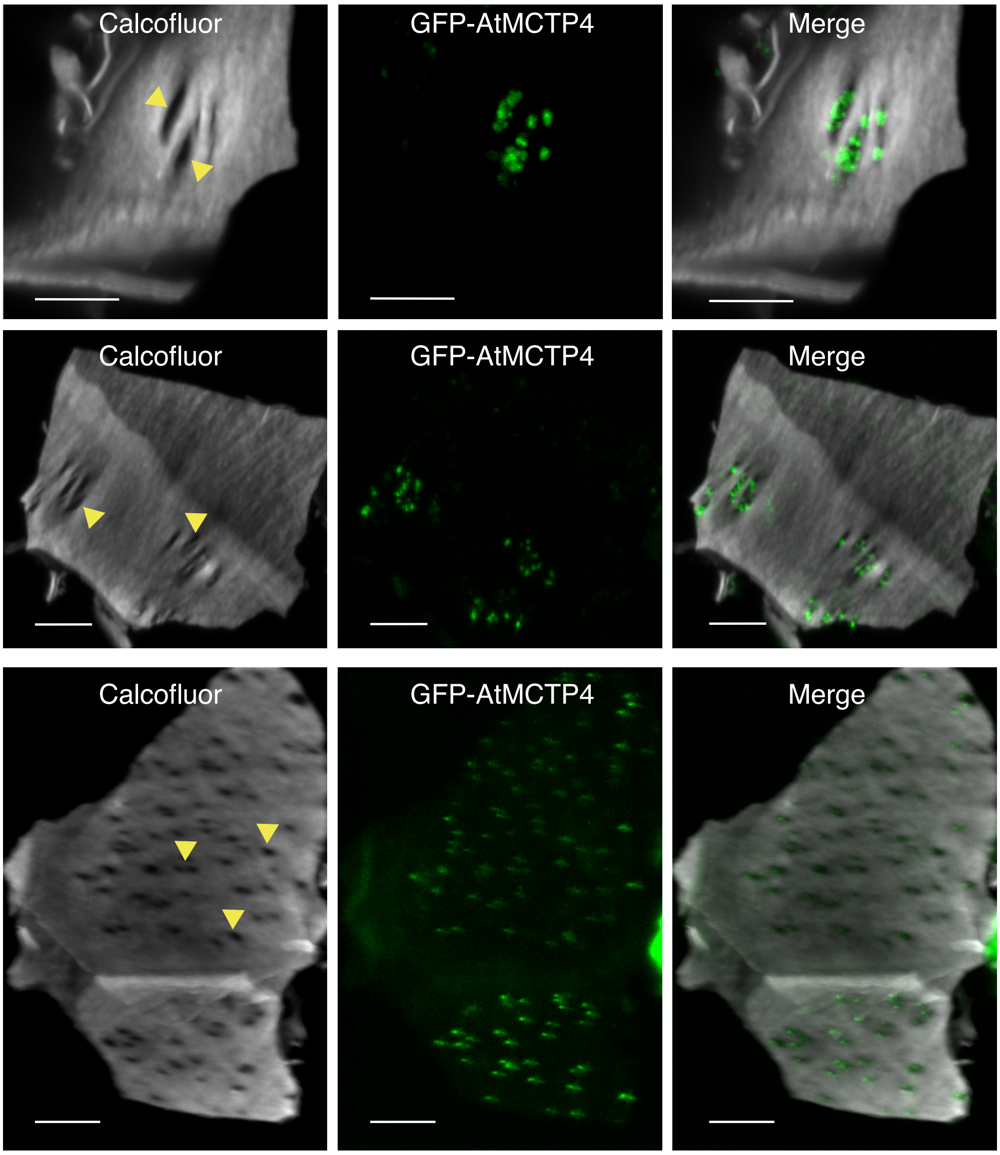

Appendix Figure S10

Confocal observation of cell walls purified from pAtMCTP4:GFP-AtMCTP4. Cell walls were stained with calcofluor, revealing plasmodesmata pit fields where calcofluor staining, hence cellulose, is absent/reduced (yellow arrowheads). GFP-AtMCTP4 signal is always associated with plasmodesmata pit fields. Scale bars, 5 µm

Appendix Figure S11

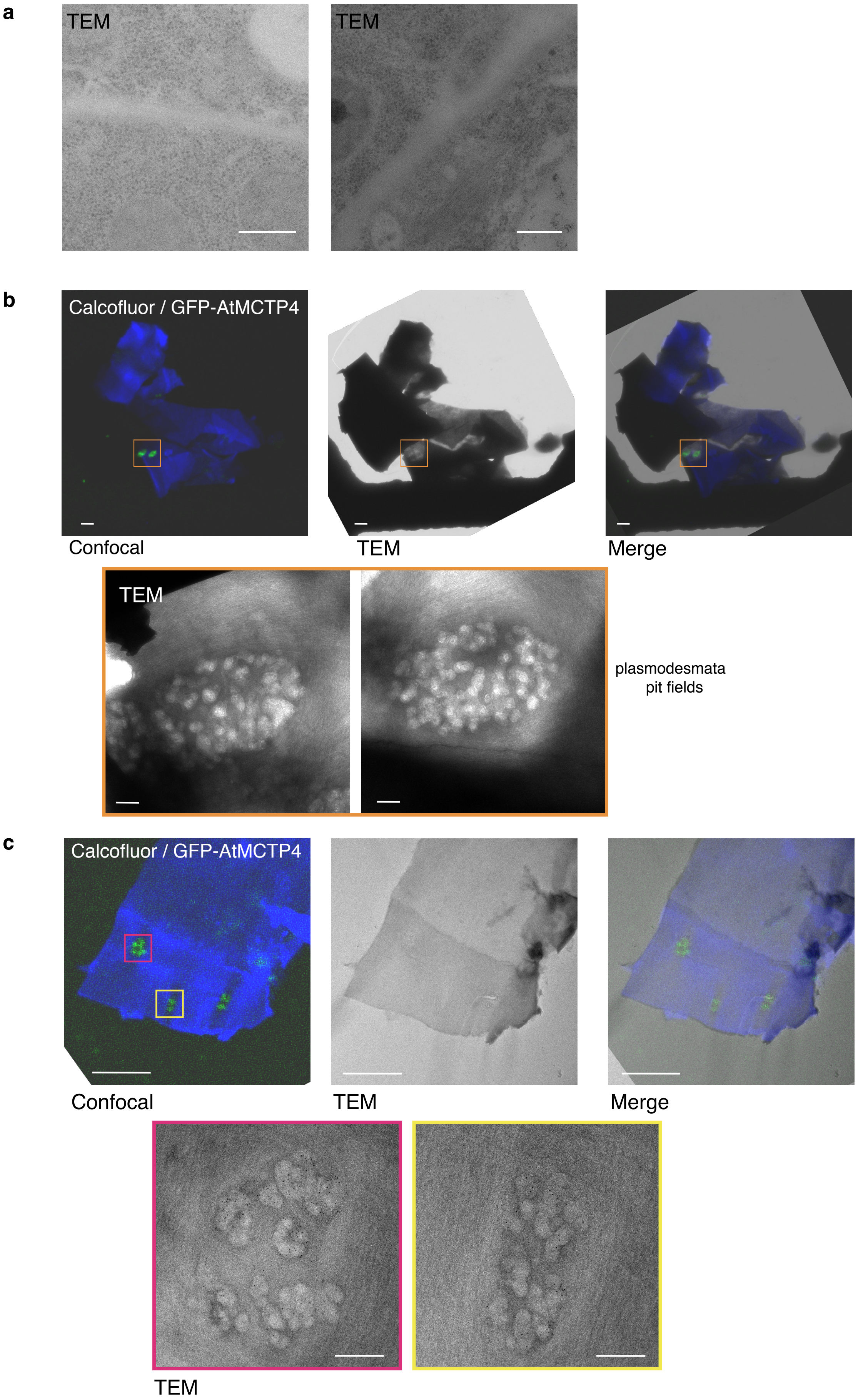

Appendix Figure S11

(a) Immunogold labelling for GFP on Col-0 wild type roots. Scale bar, 300 nm. (b) CLEM on cell walls purified from pAtMCTP4:GFP-AtMCTP4 *Arabidopsis* seedlings (c) CLEM combined with immunogold labelling against callose (10 nm gold particles). TEM = transmission electron microscopy. Scale bars, 5 µm for confocal images and 300 nm for TEM images.

Appendix Figure S12

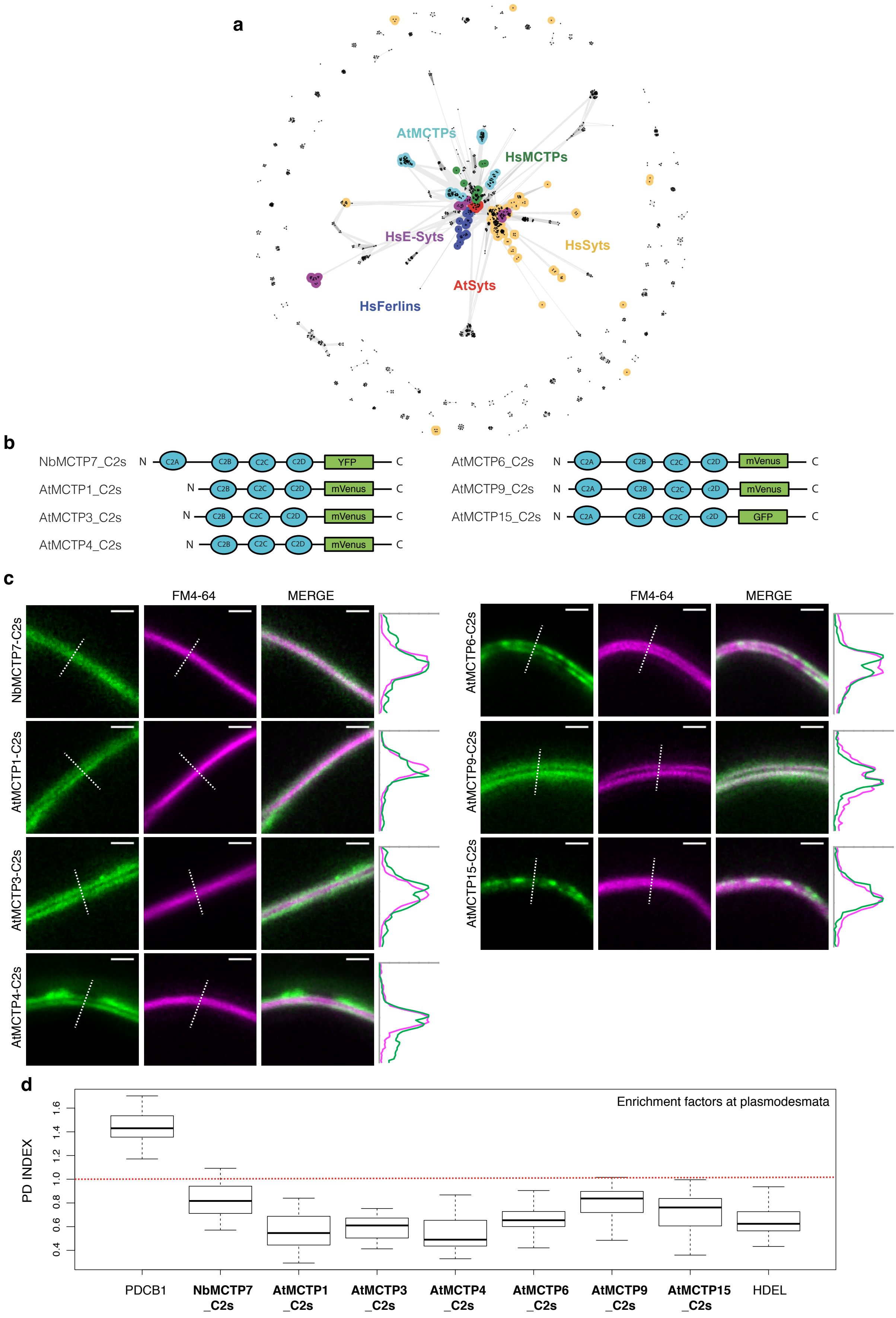

Appendix Figure S12

(a) Cluster map of human and *A. thaliana* C2 domains. Homologs of the four *A. thaliana* MCTP C2 domains were searched for in the human and *A. thaliana* proteomes using HHpred with a probability cut-off of 50% and with 'No. of target sequences' set to 10000. The obtained sequences were filtered to a maximum pairwise sequence identity of 100%, at a length coverage of 70%, using MMseqs2 (cite PMID: 29035372) to eliminate redundant sequences. The sequences in the filtered set, comprising almost all human and *A. thaliana* C2 domains, were next clustered in CLANS based on their all-against-all pairwise sequence similarities as evaluated by BLAST P-values (PMID: 9254694). Clustering was done to equilibrium in 2D at a P-value cutoff of e-10 using default settings. In the map, dots represent sequences and line coloring reflects the strength of sequence similarity between them; the darker a line, the lower the P-value. Proteins not discussed in the manuscript are not colored.

(b-d) The C2 blocks (C2A-D or C2B-D) of AtMCTP1, 3, 4, 6, 9, 15 and NbMCTP7 were tagged at their C-terminus with a fluorescent tag and expressed transiently in *N. benthamiana* leaves under moderate ubiquitin 10 promoter. b, Schematic representation of truncated MCTPs tagged with a fluorescent tag. c, Localisation of truncated AtMCTP1, 3, 4, 6, 9, 15 and NbMCTP7 C2 blocks (MCTP-C2s) in *N. benthamiana* epidermal cells by confocal microscopy. The PM was stained using short-term (up to 15 min) FM4-64 staining (magenta). Intensity plots are shown for each co-localisation pattern. When expressed in epidermal cells, MCTP-C2s-YFP constructs only partially associate with the PM and cytosolic localisation is also apparent. Scale bars, 5 µm. d, The PD index of individual truncated MCTP_C2s constructs is below 1 (red dashed line), indicating loss of plasmodesmata localisation. In the box plot, median value is represented by horizontal line, values between quartil 1 to 3 are represented by box ranges, minimum and maximum values are represented by error bars. 3 biological replicates were analysed.

Appendix Figure S13

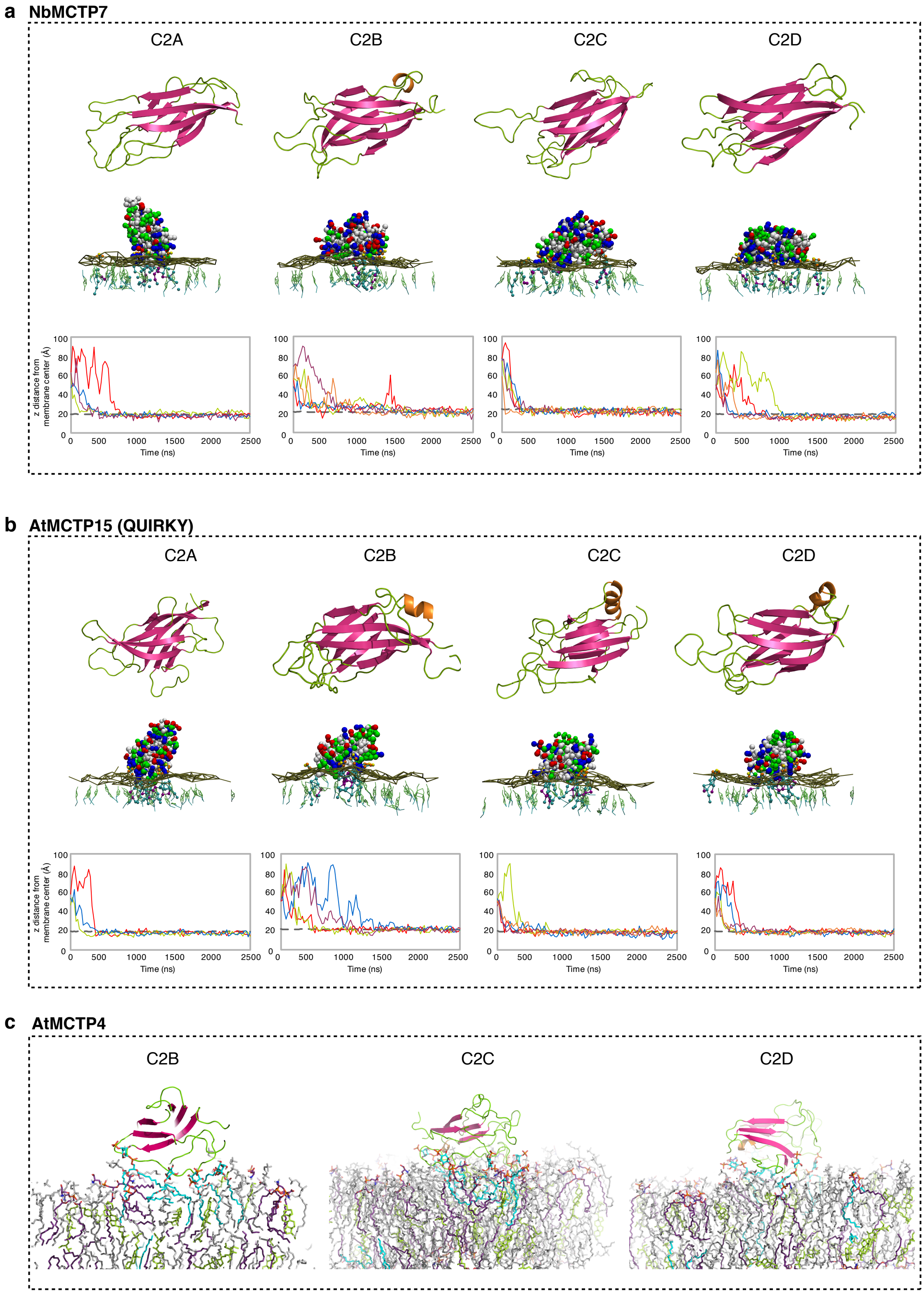

Appendix Figure S13

Membrane docking of NbMCTP7 and AtMCTP15/QKY C2 domains on a PM-like membrane.

In (a) and (b); Top: 3D-atomistic model of the individual AtMCTP4 C2 domains. Beta strands are shown in pink, loops in green and alpha helices in orange. Bottom: molecular dynamics of individual NbMCTP7 (a) and AtMCTP15/QKY (b) C2 domains with phosphatidylcholine (PC), phosphatidylserine (PS), sitosterol (Sito) and phosphoinositol-4-phosphate (PI4P) (PC/PS/Sito/PI4P 57:19:20:4) biomimetic lipid bilayer. The plots show the minimal distance between the protein's closest residue to the membrane and the membrane center, over time. The membrane's phosphate plane is represented by a PO_4 grey line on the graphs and a dark green meshwork on the simulation image captures (above graphs). For individual C2 domain, the simulations were repeated three to five times (runs 1-5). C2 membrane docking was only considered as positive when a minimum of three independent repetitions showed similarly stable interaction with the membrane. All C2 domains of NbMCTP7 and AtMCTP15/QKY show membrane interaction with a "PM-like" membrane composition, mainly due to the presence of PI4P. The amino acid colour code is as follow: red, negatively charged (acidic) residues; blue, positively charged (basic) residues; green, polar uncharged residues; and white, hydrophobic residues. (c) All atom simulation of AtMCTP4 C2 domains. 3D representation of the AtMCTP4 C2 domains still interacting with lipids after 100 ns all atom simulations. The C2 domain color code used is the same as in (a). PC is represented in grey, PS in purple, sitosterol in green and PI4P in blue.

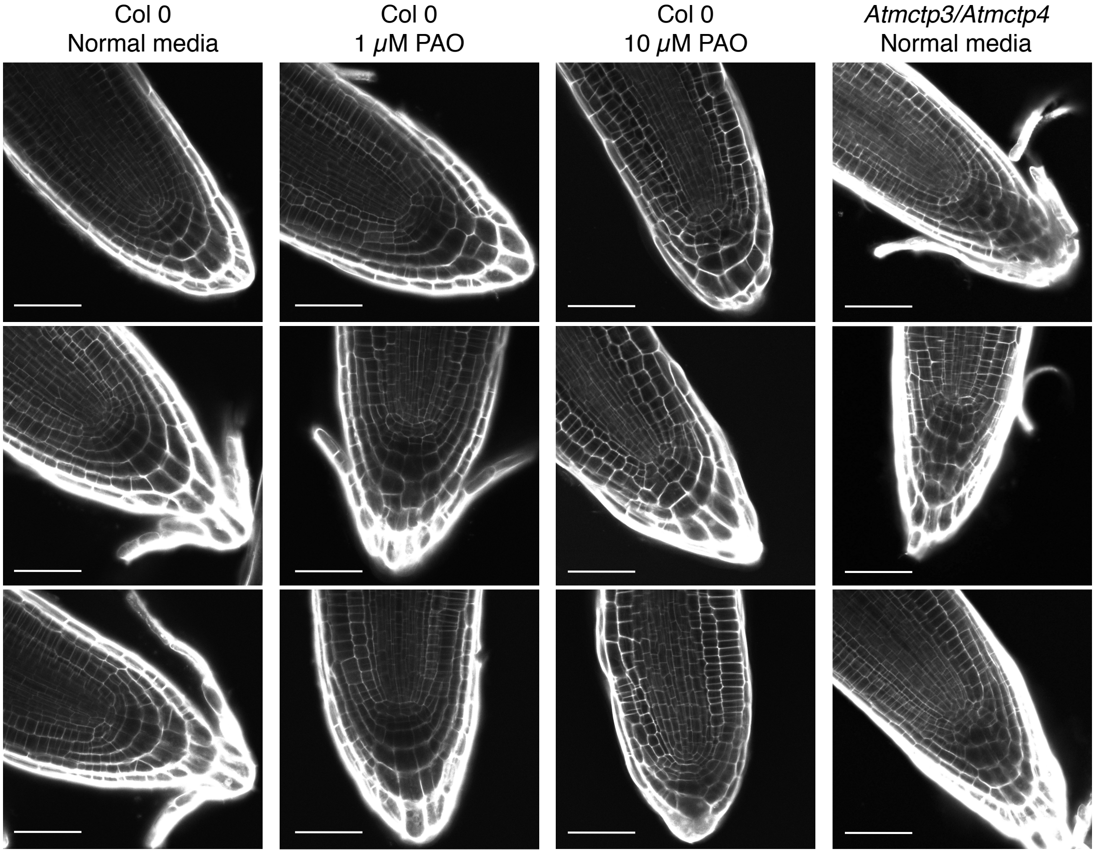

Appendix Figure S14

PAO treated Col-0 *Arabidopsis* seedlings. After 7 days on MS solid media containing 1 μM or 10 μM PAO, root organisation was visualised by propidium iodide staining. At 1 μM PAO cell organisation at the root tip was aberrant in 4 out of 12 plants against 12 out of 12 in 10 μM PAO conditions. Scale bar, 50μm.

Appendix Table S1

Candidat number	Primary Accession	Secondary Accessions	Description	Abundance	Enrichment ratios				Presence in published ER proteomes		PD association in *Arabidopsis* references
					PD/PM	PD/TP	PD/μ	PD/CW	Nikolovski et al. 1	Dunkley et al. 2	
1	AT1G51570.1	AT1G51570.1 ;AT1G04150.1 ;AT3G03680.1 ;AT5G43740.1 ;AT5G44760.1	**Multiple C2 domains and Transmembrane region Protein 4,10,14 (MCTP4,10,14)**	2093561645	351.0	223.6	360.1	70.2	×	×	
2	AT5G42100.2	AT5G42100.2 ;AT5G42100.1	Beta-1-3-glucanase (AtBG_PAPP)	1638015771	164.0	247.2	580.8	45.0			3
3	AT4G16380.1	AT4G16380.1 ;AT4G16380.2 ;AT4G16380.3 ;AT4G16380.4	Heavy metal transport/detoxification superfamily protein	1355301110	1022.7	478.1	1318.4	72.8			
4	AT5G62890.1	AT5G62890.1 ;AT5G62890.4	Xanthine/uracil permease family protein (AtNAT6)	1135513188	772.6	730.3	1308.9	96.0	×		
5	AT1G22610.1	AT1G22610.1 ;AT4G00700.1	**Multiple C2 domains and Transmembrane region Protein 6,9 (MCTP6,9)**	776007012	315.5	115.1	285.3	61.7			
6	AT3G52470.1	AT3G52470.1 ;AT2G35980.1 ;AT5G06330.1	Late embryogenesis abundant (LEA) hydroxyproline-rich glycoprotein family	643353656	123.3	137.5	323.3	97.4			
7	AT5G16510.1	AT5G16510.1	Alpha-1-4-glucan-protein synthase family protein	494288348	661.2	206.1	772.3	886.8			
8	AT5G61130.1	AT5G61130.1	Plasmodesmata callose-binding protein 1 (PDCB1)	328259264	219.2	1052.3	623.0	48.0			4
9	AT5G43980.1	AT5G43980.1	Plasmodesmata-located protein 1 (PDLP1)	311480268	309.0	119.0	307.6	46.4			5
10	AT2G01820.1	AT2G01820.1	Leucine-rich repeat protein kinase family protein (TMK3)	285991310	28.9	60.4	137.5	241.7			
11	AT5G13000.1	AT5G13000.1 ;AT3G14570.1 ;AT3G14570.2 ;AT3G14570.3 ;AT3G14780.1 ;AT5G13000.2	Glucan synthase-like 12 (CALS3)	257637656	14.5	56.4	67.3	65.2			6
12	AT5G06320.1	AT5G06320.1	NDR1/HIN1-like 3 (NHL3)	251025320	47.8	104.2	95.4	41.6			
13	AT3G51740.1	AT3G51740.1 ;AT3G56100.1	Inflorescence meristem receptor-like kinase 2 (IMK2)	245842528	17.5	43.5	57.1	52.5			
14	AT2G01630.1	AT2G01630.1 ;AT2G01630.2 ;AT2G01630.3	O-Glycosyl hydrolases family 17 protein (beta1-3 glucanase, PdBG2)	232481254	26.9	73.3	89.6	48.4			7
15	AT5G48450.1	AT5G48450.1 ;AT5G48450.2	SKU5 similar 3	204842485	62.4	42.9	75.0	52.7			
16	AT5G46700.1	AT5G46700.1	Tetraspanin family protein (TRN2, TET1)	190712794	92.2	278.8	253.6	120.1			
17	AT1G60030.1	AT1G60030.1	Nucleobase-ascorbate transporter 7 (AtNAT7)	175342944	228.0	548.4	468.1	42.0			
18	AT2G01660.1	AT2G01660.1 ;AT2G01660.2 ;AT2G01660.3	Plasmodesmata-located protein 6 (PDLP6)	159384568	193.7	126.1	637.9	52.3			5
19	AT2G25270.1	AT2G25270.1	Transmembrane protein	139593159	152.4	156.1	198.2	74.8			
20	AT1G32090.1	AT1G32090.1 ;AT1G62320.1 ;AT1G62320.2 ;AT1G62320.3 ;AT1G62320.4	Early-responsive to dehydration stress protein (ERD4)	111499705	17.7	40.0	82.5	68.4			
21	AT1G18650.1	AT1G18650.1 ;AT1G18650.2	Plasmodesmata callose-binding protein 3 (PDCB3)	100145419	101.4	63.2	76.5	46.8			4
22	AT2G23810.1	AT2G23810.1	Tetraspanin 8 (TET8)	97572093	98.9	73.8	180.3	60.9			
23	AT3G11660.1	AT3G11660.1	NDR1/HIN1-like 1 (NHL1)	83423848	57.1	62.0	70.9	77.1			
24	AT3G54200.1	AT3G54200.1	Late embryogenesis abundant (LEA) hydroxyproline-rich glycoprotein family	81984458	16.9	197.0	89.5	48.8			
25	AT4G29360.1	AT4G29360.1 ;AT4G29360.2	O-Glycosyl hydrolases family 17 protein	79706419	68.0	70.0	155.6	47.5			
26	AT1G69295.1	AT1G69295.1 ;AT1G26450.1 ;AT1G69295.2	Plasmodesmata callose-binding protein 4 (PDCB4)	79562157	107.9	133.1	129.2	47.5			4
27	AT5G63530.2	AT5G63530.2 ;AT5G63530.1	Farnesylated protein 3	76940500	153.5	83.4	157.5	41.7			
28	AT1G66250.1	AT1G66250.1	O-Glycosyl hydrolases family 17 protein (beta1-3 glucanase, PdBG3)	71917917	32.9	204.2	237.4	59.5			7
29	AT2G33330.1	AT2G33330.1	Plasmodesmata-located protein 3 (PDLP3)	71730983	251.4	90.8	325.4	60.7			5
30	AT1G32060.1	AT1G32060.1	Phosphoribulokinase	71318252	19.8	76.5	134.0	780.2			
31	AT2G05760.1	AT2G05760.1	Xanthine/uracil permease family protein (AtNAT1)	69482506	274.8	452.3	702.1	46.0			
32	AT4G34150.1	AT4G34150.1	Calcium-dependent lipid-binding family protein Soc C-term domain charged block	67987664	270.5	32.9	225.9	178.7			
33	AT2G26510.1	AT2G26510.1 ;AT2G26510.2 ;AT2G26510.3	Xanthine/uracil permease family protein (AtNAT3)	66876643	87.1	54.6	41.8	67.5			
34	AT2G12400.1	AT2G12400.1	Plasma membrane fusion protein	66330049	25.9	80.9	107.0	91.4			
35	AT5G49990.1	AT5G49990.1	Xanthine/uracil permease family protein (AtNAT4)	66145014	112.7	171.7	277.7	77.3			
36	AT3G13560.1	AT3G13560.1	O-Glycosyl hydrolases family 17 protein (beta1-3 glucanase, PdBG1)	65897722	42.7	148.4	287.3	52.3			7
37	AT1G64760.1	AT1G64760.1 ;AT3G04010.1 ;AT5G18220.1	O-Glycosyl hydrolases family 17 protein	64825238	12.3	63.2	48.9	38.1			
38	AT5G58090.1	AT5G58090.1	O-Glycosyl hydrolases family 17 protein	62685626	27.8	88.9	96.6	45.0			
39	AT2G31810.1	AT2G31810.1 ;AT2G31810.2 ;AT2G31810.3	ACT domain-containing small subunit of acetolactate synthase protein	61594943	74.3	34.3	223.6	46.6			
40	AT4G25240.1	AT4G25240.1	SKU5 similar 1	58538694	17.3	61.9	94.1	60.8			
41	AT2G36850.1	AT2G36850.1	Glucan synthase-like 8 (CALS10, GSL8)	52456367	8.0	20.8	26.4	23.9			
42	AT3G45600.1	AT3G45600.1 ;AT3G45600.2 ;AT5G60220.1	Tetraspanin 3 (TET3)	47760446	65.3	102.4	242.7	51.0			10
43	AT5G61030.1	AT5G61030.1	Glycine-rich RNA-binding protein 3	45152284	185.8	40.8	211.6	189.0			
44	AT1G69700.1	AT1G69700.1	HVA22 homologue C	43597164	222.7	137.1	204.2	86.5			
45	AT2G17120.1	AT2G17120.1	LysM domain-containing GPI-anchored protein 2 (LYM2)	40630549	2.7	18.3	10.3	35.9			8
46	AT3G53780.2	AT3G53780.2 ;AT3G53780.1 ;AT3G53780.3	RHOMBOID-like protein 4	40497867	103.2	104.2	195.7	57.7			
47	AT1G04520.1	AT1G04520.1	Plasmodesmata-located protein 2 (PDLP2)	38475248	172.0	78.7	74.5	44.9			5
48	AT4G31140.1	AT4G31140.1 ;AT5G20870.1	O-Glycosyl hydrolases family 17 protein	36693093	21.7	75.7	127.0	48.3			
49	AT3G11650.1	AT3G11650.1	NDR1/HIN1-like 2 (NHL2)	34803434	308.4	96.8	306.7	50.3			
50	AT1G04040.1	AT1G04040.1 ;AT5G44020.1	HAD superfamily subfamily IIIB acid phosphatase	33114051	292.5	119.0	264.1	376.2			
51	AT5G61730.2	AT5G61730.2 ;AT5G61690.1 ;AT5G61690.2 ;AT5G61730.1	ABC2 homolog 11	32595047	10.7	36.3	51.4	83.7			
52	AT1G05570.1	AT1G05570.1 ;AT1G05570.2 ;AT1G06490.1 ;AT1G06490.2	Callose synthase 1 (CALS1, GSL6)	29840182	14.0	39.5	40.0	69.2			
53	AT5G61740.1	AT5G61740.1 ;AT3G47740.1 ;AT3G47750.1 ;AT3G47760.1 ;AT3G47760.2 ;AT3G47760.3 ;	ABC2 homolog 14	26706448	9.6	43.5	63.1	69.4			
54	AT4G35060.1	AT4G35060.1	Heavy metal transport/detoxification superfamily protein	26431305	20.7	136.7	137.9	57.9			
55	AT2G35960.1	AT2G35960.1	NDR1/HIN1-like 12 (NHL12)	25592331	196.4	46.6	186.0	66.5			
56	AT5G17980.1	AT5G17980.1	**Multiple C2 domains and Transmembrane region Protein 16 (MCTP16)**	23482273	59.7	33.5	126.7	34.9	×	×	
57	AT2G38010.1	AT2G38010.1 ;AT1G07380.1 ;AT1G07380.2 ;AT2G38010.2 ;AT2G38010.3	Neutral/alkaline non-lysosomal ceramidase	22829457	107.4	56.4	68.5	44.4			
58	AT1G74010.1	AT1G74010.1 ;AT5G56380.1	Calcium-dependent phosphotriesterase superfamily protein	21457306	133.6	81.0	207.8	75.2			
59	AT5G03300.1	AT5G03300.1 ;AT5G03300.2	Adenosine kinase 2	21310606	185.4	34.8	149.9	88.8			
60	AT3G57880.1	AT3G57880.1 ;AT4G11610.1 ;AT4G11610.2 ;AT4G11610.3	**Multiple C2 domains and Transmembrane region Protein 3, 7 (MCTP3, 7)**	20441820	47.5	44.3	96.9	81.7	×	×	
61	AT2G01080.1	AT2G01080.1	Late embryogenesis abundant (LEA) hydroxyproline-rich glycoprotein family	20172878	91.7	33.8	112.7	45.3			
62	AT5G15400.1	AT5G15400.1	U-box domain-containing protein	18414944	237.2	33.9	256.6	232.3			
63	AT2G42010.1	AT2G42010.1 ;AT2G42010.2 ;AT4G00240.1 ;AT4G00240.2 ;AT4G00240.3 ;AT4G11830.1 ;	Phospholipase D beta 1 (PLDBETA1)	18265056	189.7	46.1	149.7	35.4			
64	AT1G23880.1	AT1G23880.1 ;AT1G23880.2	NHL domain-containing protein	16952398	186.3	52.5	134.8	107.1			
65	AT1G08210.1	AT1G08210.1 ;AT1G08210.2 ;AT1G08210.3 ;AT1G08210.4	Eukaryotic aspartyl protease family protein	15438618	162.3	132.8	172.9	50.4			
66	AT1G74720.1	AT1G74720.1	**Multiple C2 domains and Transmembrane region Protein 15 (MCTP5, QUIRKY, QKY**	15148937	79.0	47.9	82.9	73.1			11
67	AT5G67130.1	AT5G67130.1	PLC-like phosphodiesterases superfamily protein	15053353	22.9	43.0	64.9	52.1			
68	AT5G55050.1	AT5G55050.1	GDSL-like Lipase/Acylhydrolase superfamily protein	14212548	267.9	56.2	236.3	83.1			
69	AT1G64450.1	AT1G64450.1	Glycine-rich protein family	14151663	201.6	104.5	418.2	93.4			
70	AT1G74520.1	AT1G74520.1	HVA22 homologue A	14058943	144.1	36.6	76.2	78.5			
71	AT4G25550.1	AT4G25550.1	Cleavage/polyadenylation specificity factor 25kDa subunit	12069569	553.7	56.8	222.5	41.0			
72	AT2G20850.1	AT2G20850.1 ;AT2G20850.2	STRUBBELIG-receptor family 1 (SFR1)	11280786	8.0	60.7	63.0	60.8			
73	AT5G12970.1	AT5G12970.1	**Multiple C2 domains and Transmembrane region Protein 5 (MCTP5)**	9974540	102.5	516.4	171.4	152.6			
74	AT4G05520.1	AT4G05520.1 ;AT4G05520.2	EPS15 homology domain 2	9223837	24.2	63.0	91.8	57.6			
75	AT4G04970.1	AT4G04970.1 ;AT4G13690.1	Glucan synthase-like 1 (CALS11, GSL1)	8134750	53.8	55.1	93.8	90.2			
76	AT2G27810.1	AT2G27810.1 ;AT2G27810.2 ;AT2G27810.3 ;AT2G27810.4	Nucleobase-ascorbate transporter 12 (AtNAT12)	8121544	18.2	50.9	109.0	57.2			
77	AT1G73590.1	AT1G73590.1	Auxin efflux carrier family protein	7752624	12.8	160.1	59.8	156.8			
78	AT2G31960.1	AT2G31960.1 ;AT2G13680.1 ;AT2G31960.4	Glucan synthase-like 3 (CALS2, GSL3)	7719612	24.4	81.2	57.1	88.2			
79	AT2G27080.1	AT2G27080.1	Late embryogenesis abundant (LEA) hydroxyproline-rich glycoprotein family	7372898	128.1	106.0	288.0	89.7			
80	AT1G11130.1	AT1G11130.1 ;AT1G11130.2	Leucine-rich repeat protein kinase family protein (SUB)	6660962	31.8	40.7	68.3	55.6			11
81	AT3G17350.1	AT3G17350.1 ;AT3G17350.2	Wall-associated receptor kinase carboxy-terminal protein	6495607	48.3	38.0	86.7	55.9			
82	AT4G27080.1	AT4G27080.1 ;AT3G20560.1 ;AT4G27080.2	PDI-like 5-4	6261964	327.2	121.2	61.0	648.1	×		
83	AT5G07250.2	AT5G07250.2 ;AT5G07250.1	RHOMBOID-like protein 3	5887061	27.6	81.9	296.7	101.2			
84	AT4G25810.1	AT4G25810.1	Xyloglucan endotransglycosylase 6	5353773	815.6	77.1	361.2	40.8			
85	AT3G60320.1	AT3G60320.1	bZIP domain class transcription factor	5213727	83.5	51.9	156.7	92.9			
86	AT2G21185.1	AT2G21185.1	Transmembrane protein	5171328	977.8	69.7	1668.4	158.8			
87	AT4G01410.1	AT4G01410.1	Late embryogenesis abundant (LEA) hydroxyproline-rich glycoprotein family	4702568	132.6	151.5	600.1	61.1			
88	AT1G14340.1	AT1G14340.1	RNA-binding (RRM/RBD/RNP motifs) family protein	4578430	61.2	75.6	103.3	67.4			
89	AT3G56640.1	AT3G56640.1	Exocyst complex component (SEC15A)	4425140	31.8	53.1	175.4	161.5			
90	AT4G36860.3	AT4G36860.3 ;AT4G36860.1 ;AT4G36860.2	LIM domain-containing protein	4416701	83.4	40.8	62.2	44.2			
91	AT1G10180.1	AT1G10180.1	LOW protein: exocyst complex component-like protein	3755718	26.8	37.2	126.3	212.4			
92	AT4G35730.1	AT4G35730.1 ;AT1G25420.1 ;AT1G25420.4 ;AT2G21340.1 ;AT2G21340.2	Regulator of Vps4 activity in the MVB pathway protein	3709271	98.1	77.6	83.1	81.1			
93	AT1G71890.1	AT1G71890.1	Major facilitator superfamily protein (SUC5)	3477606	12.5	155.7	126.4	43.1			
94	AT4G24610.2	AT4G24610.2 ;AT4G24610.1 ;AT4G24610.3 ;AT4G24610.4	Pesticidal crystal cry8Ba protein	2983389	21.9	54.5	218.2	304.9			
95	AT5G13760.1	AT5G13760.1	Plasma-membrane choline transporter family protein (MXE10.1)	2891632	22.1	59.6	81.4	82.2			
96	AT1G14870.1	AT1G14870.1	Plant cadmium resistance 2	2883757	11.9	100.6	217.7	84.7			
97	AT1G01540.2	AT1G01540.2 ;AT1G01540.1	Protein kinase superfamily protein	2522538	15.0	35.8	71.8	87.3			
98	AT4G31540.1	AT4G31540.1	Exocyst subunit exo70 family protein G1	2492809	23.5	34.8	84.6	59.9			
99	AT3G60720.1	AT3G60720.1 ;AT3G60720.2 ;AT3G60720.3	Plasmodesmata-located protein 8 (PDLP8)	2101866	365.8	32.1	214.6	48.5			5
100	AT5G58300.1	AT5G58300.1	Leucine-rich repeat protein kinase family protein	1987533	25.5	72.9	56.9	67.2			
101	AT5G58600.1	AT5G58600.1 ;AT5G58600.2	Pmr5/Cas1p GDSL/SGNH-like acyl-esterase family protein	1961558	145.0	74.7	264.3	63.3			
102	AT4G23470.1	AT4G23470.1 ;AT4G23470.2 ;AT4G23470.3 ;AT4G23470.4	PLAC8 family protein	1700848	12.4	177.8	699.1	259.3			
103	AT1G70280.1	AT1G70280.1 ;AT1G70280.2	NHL domain-containing protein	1681602	1147.4	43.6	298.8	103.6			
104	AT1G11440.1	AT1G11440.1	Hypothetical protein	1502604	146.4	45.0	52.2	30.1			
105	AT5G19080.1	AT5G19080.1	RING/U-box superfamily protein	1275986	123.5	118.6	857.6	197.2			
106	AT4G03210.1	AT4G03210.1 ;AT4G03210.2	Xyloglucan endotransglucosylase/hydrolase 9	1260364	77.3	81.5	154.0	68.8			
107	AT1G22090.1	AT1G22090.1	Hypothetical protein	1093393	174.2	230.4	45.8	33.0			
108	AT1G02730.1	AT1G02730.1	Cellulose synthase-like D5	1076244	57.5	37.9	101.0	200.5			
109	AT5G11850.1	AT5G11850.1	Protein kinase superfamily protein	975840	14.3	50.9	767.6	57.1			
110	AT2G40815.1	AT2G40815.1 ;AT2G40815.2	Calcium-dependent lipid-binding family protein	971928	51.4	424.6	12960.5	204.1			
111	AT3G09770.1	AT3G09770.1 ;AT3G09770.2	RING/U-box superfamily protein	727501	23.5	782.6	170.4	75.2			
112	AT3G15720.4	AT3G15720.4 ;AT3G15720.1 ;AT3G15720.2 ;AT3G15720.3	Pectin lyase-like superfamily protein	656886	415.8	491.5	471.5	919.5			
113	AT1G71040.1	AT1G71040.1	Cupredoxin superfamily protein	608171	361.4	64.2	92.1	57.9			
114	AT5G04850.1	AT5G04850.1 ;AT5G04850.2	SNF7 family protein	543337	164.5	119.1	163.8	79.2			
115	AT4G14130.1	AT4G14130.1	Xyloglucan endotransglucosylase/hydrolase 15	535716	384.7	81.8	211.0	111.5			

References

1 Nikolovski, N. *et al.* Putative glycosyltransferases and other plant golgi apparatus proteins are revealed. *Plant Physiol.* **160,** 1037–1051 (2012).
2 Dunkley, T. P. J. *et al.* Mapping the Arabidopsis organelle proteome. *PNAS* **103,** 6518–6523 (2006).
3 Levy, A., Erlanger, M., Rosenthal, M. & Epel, B. L. A plasmodesmata-associated beta-1,3-glucanase in Arabidopsis. *Plant J.* **49,** 669–682 (2007).
4 Simpson, C., Thomas, C., Findlay, K., Bayer, E. & Maule, A. J. An Arabidopsis GPI-anchor plasmodesmal neck protein with callose binding activity and potential to regulate cell-to-cell trafficking. *Plant Cell* **21,** 581–594 (2009).
5 Thomas, C. L., Bayer, E. M., Ritzenthaler, C., Fernandez-Calvino, L. & Maule, A. J. Specific targeting of a plasmodesmal protein affecting cell-to-cell communication. *PLoS Biol.* **6,** 0180–0190 (2008).
6 Vatén, A. *et al.* Callose biosynthesis regulates symplastic trafficking during root development. *Dev. Cell* **21,** 1144–1155 (2011).
7 Benitez-Alfonso, Y. *et al.* Symplastic intercellular connectivity regulates lateral root patterning. *Dev. Cell* **26,** 136–147 (2013).
8 Faulkner, C. *et al.* LYM2-dependent chitin perception limits molecular flux via plasmodesmata. *Proc. Natl. Acad. Sci. U. S. A.* **110,** 9166–70 (2013).
9 Cui, W. & Lee, J.-Y. Arabidopsis callose synthases CalS1/8 regulate plasmodesmal permeability during stress. *Nat. Plants* **2,** 16034 (2016).
10 Fernandez-Calvino, L. *et al.* Arabidopsis plasmodesmal proteome. *PLoS One* **6,** e18880 (2011).
11 Vaddepalli, P. *et al.* The C2-domain protein QUIRKY and the receptor-like kinase STRUBBELIG localize to plasmodesmata and mediate tissue morphogenesis in Arabidopsis thaliana. *Development* **141,** 4139–4148 (2014).

Appendix Table S1. Proteins of the core *Arabidopsis* plasmodesmata proteome

Label-free quantitation strategy was used to determine the relative abundance of proteins in the plasmodesmata (PD) fraction *versus* contaminant subcellular fractions namely, the PM, total extract (TP), microsomes (μ) and cell wall (CW), see Methods for details. Only proteins presenting minimum enrichment ratios of 8, 40, 30 and 30 in plasmodesmata *versus* PM, TP, microsomal and CW fractions, respectively were selected. Previously characterised plasmodesmal proteins are in orange and MCTP members in green. First row indicates the main accession and second row all possible isoforms potentially identified. The different shades (light to dark) of brown represent different enrichment levels (0-10; 10-20; 20-100 and above 100).

Appendix Table S2
Primers used for MCTP cloning.

CLONING					
**MCTP Full lenght**	**construction**	**plasmid**		**primers Forward/ right border**	**primers Reverse / left border**
NbMCTP7	p35S:GFP:NbMCTP7	pGWB406		**GGGGACAAGTTTGTACAAAAAAGCAGGctta**ATGATCTTAAGTAATCTGAAGCTAGGTGTCG	**GGGGACCACTTTGTACAAGAAAGCTGGG**TTACAACATACTATCTGTTCGAGCAGGAAG
AtMCTP3	pUBQ10:eYFP:AtMCTP3	pK7m34GW		**GGGGACAGCTTTCTTGTACAAAGTGGaa**ATGCAGAGACCACCTCCTGAAG	**GGGGACAACTTTGTATAATAAAGTTGaCTA**CCAAAAACACAAACTTATCTTAC
AtMCTP4	promAtMCTP4:GFP:AtMCTP4	pRBbar-OCS	GFP-AtMCTP4	CGTCGAGCAAGGATCCATGGTGAGCAAGGGCGAGGA	AAAGCAGGGCATGCCTCAGAGCATGCAATCAGTTCTTGCT
			promAtMCTP4	GCTCACTAGTGAATTCTCCACCTTTCCCAATCAAGCTTCCA	TAACATTGTTGGATCCTTGCTCGACGAGGTCATATGTGGTT
AtMCTP4	pUBQ10:eYFP:AtMCTP4	pB7m34GW		**GGGGACAGCTTTCTTGTACAAAGTGGaa**ATGCAGAGACCACCTCCTGAAG	**GGGGACAACTTTGTATAATAAAGTTGaCTA**CCAAAAACACAAACTTATCTTAC
AtMCTP6	p35S:eGFP:AtMCTP6	pB7WGF2		caccATGAATAAACTAGTTGTAGAAATC	TTACAGTAGCATATCTGACTTGGC
AtMCTP9	pUBQ10:eYFP:AtMCTP9	pB7m34GW		**GGGGACAGCTTTCTTGTACAAAGTGGaa**ATGAGCAATATAAAGCTAGGA	**GGGGACAACTTTGTATAATAAAGTTGa**TCACAGCATAGAGTCGGTCATG
**MCTP_TMR**					
NbMCTP7_TMR	p35S:GFP:NbMCTP7 TMD	pK7WGY2		**GGGGACAAGTTTGTACAAAAAAGCAGGCTtaATG**TCGTTTGCGAACATGCTTTA	**GGGGACCACTTTGTACAAGAAAGCTGGGTt**TTACAACATACTATCTGTTCG
ATMCTP1_TMR	pUBQ10:eYFP:AtMCTP1 TMD	pB7m34GW		**GGGGACAGCTTTCTTGTACAAAGTGGaaATG**CTTGTTCTGCAAACAAAA	**GGGGACAACTTTGTATAATAAAGTTGa**TCAAAGCATACAATCTGCTTTTGA
ATMCTP3_TMR	pUBQ10:eYFP:AtMCTP3 TMD	pB7m34GW		**GGGGACAGCTTTCTTGTACAAAGTGGaa**ATGCTTGATGTGGGTTCTCAC	**GGGGACAACTTTGTATAATAAAGTTGt**CTATCAGAGCATGCAATCAGTTC
ATMCTP4_TMR	pUBQ10:eYFP:AtMCTP4 TMD	pK7m34GW		**GGGGACAGCTTTCTTGTACAAAGTGGaa**ATGCGTAGAAGCAAGGCGAAT	**GGGGACAACTTTGTATAATAAAGTTGa**CTATCAGAGCATGCAATCAGTTCT
ATMCTP6_TMR	pUBQ10:eYFP:AtMCTP6 TMD	pB7m34GW	*synthesis & cloning into P2RP3 by Genescript*	AGGAGAAGCAAAGCTAATTT	AGTCAGATATGCTACTGTAA
ATMCTP9_TMR	pUBQ10:eYFP:AtMCTP9 TMD	pB7m34GW	*synthesis & cloning into P2RP3 by Genescript*	ATGGAACAAGTGTGCACATG	TGACCGACTCTATGCTGTGA
ATMCTP15_TMR	pUBQ10:eYFP:AtMCTP15 TMD	pB7m34GW	*synthesis & cloning into P2RP3 by Genescript*	AGCAAAGCGAATTGGTACAG	TGTCCGATCGACTCATCTAA
**MCTP_C2s**					
ATMCTP1_C2s	pUBQ10:AtMCTP1 C2B-D:mVenus	pB7m34GW	*synthesis & cloning into PDONR221 by Genescript*	ATGGCAGCCAAAGATGGAGC	TCACTTGTCTATCCTTGGCT
ATMCTP3_C2s	pUBQ10:AtMCTP3 C2B-D:mVenus	pB7m34GW	*synthesis & cloning into PDONR221 by Genescript*	ATGCAGAGACCACCTCCTGA	CTTGCTCTTCATTGCTCAAC
ATMCTP4_C2s	pUBQ10:AtMCTP4 C2B-D:mVenus	pB7m34GW	*synthesis & cloning into PDONR221 by Genescript*	ATGCAGAGACCACCTCCTGA	GCTCTTCATTGCTCAACATG
ATMCTP6_C2s	pUBQ10:At6 C2:mVenus	pB7m34GW		**GGGGACAAGTTTGTACAAAAAAGCAGGCTaa**ATGAATAAACTAGTTGTAGAAATCG	**GGGGACCACTTTGTACAAGAAAGCTGGGTa**GCCGGTGCAGGTGTACCTCAG
ATMCTP9_C2s	pUBQ10:At9 C2:mVenus	pB7m34GW	*synthesis & cloning into PDONR221 by Genescript*	ATGAGCAATATAAAGCTAGG	GCACATCGGTCTCCAGTATG
ATMCTP15_C2s	p35S:At15 C2 A-D:eGFP	pK7FWG2	*from C.Tréhin*		
NbMCTP7_C2s	p35S:NbMCTP7 C2A-D:eYFP	pH7YWG2		**GGGGACAAGTTTGTACAAAAAAGCAGGCTta**ATGATCTTAAGTAATCTGAAGC	**GGGGACCACTTTGTACAAGAAAGCTGGGTt**TAAAGCATGTTCGCAAACGA
**GENOTYPING**					
AtMCTP3			P1 (Fw) / P2 (Rv)	GTGGAACCAAGTTTTCGCCT	GAGAATGACTGCGGCAATCA
			P1 (Fw) / LB-GABI	GTGGAACCAAGTTTTCGCCT	ATATTGACCATCATACTCATTGC
AtMCTP4			Fw / Rv	CCTTCACCACTCTACTTCAATGT	ACCGATGTTAGGCTTCCACA
			LBb1.3-Salk / Rv	ATTTTGCCGATTTCGGAAC	ACCGATGTTAGGCTTCCACA
**Transcript expression**					
AtMCTP3				GTGGAACCAAGTTTTCGCCT	AAATTGAGAGGAACGGATGG
AtMCTP4				ATGCAGAGACCACCTCCTG	CTTGCTGGCGAATTTGAT
ACT2 (AT1G49240)				CCGAGCAGCATGAAGATTAAG	CATACTCTGCCTTAGAGATCCACA
**YEAST EXPERIMENT**					
AtMCTP4	pCU:AtMCTP4	pCU416	BamHI-AtMCTP4-Fw / SmaI-AtMCTP4stop-Rv	GGTGGTGGATCCATGCAGAGACCACCTCCTGAAG	GGTGGTCCCGGGCTATCAGAGCATGCAATCAGTTCT
